# Effect of lipid peroxidation on membrane permeability of cancer and normal cells subjected to oxidative stress†
†Electronic supplementary information (ESI) available. See DOI: 10.1039/c5sc02311d



**DOI:** 10.1039/c5sc02311d

**Published:** 2015-10-16

**Authors:** Jonas Van der Paal, Erik C. Neyts, Christof C. W. Verlackt, Annemie Bogaerts

**Affiliations:** a Research Group PLASMANT , Department of Chemistry , University of Antwerp , Universiteitsplein 1 , B-2610 Wilrijk , Antwerp , Belgium . Email: annemie.bogaerts@uantwerpen.be

## Abstract

We performed molecular dynamics simulations to investigate the effect of lipid peroxidation products on the structural and dynamic properties of the cell membrane. Our simulations predict that the lipid order in a phospholipid bilayer, as a model system for the cell membrane, decreases upon addition of lipid peroxidation products. Eventually, when all phospholipids are oxidized, pore formation can occur. This will allow reactive species, such as reactive oxygen and nitrogen species (RONS), to enter the cell and cause oxidative damage to intracellular macromolecules, such as DNA or proteins. On the other hand, upon increasing the cholesterol fraction of lipid bilayers, the cell membrane order increases, eventually reaching a certain threshold, from which cholesterol is able to protect the membrane against pore formation. This finding is crucial for cancer treatment by plasma technology, producing a large number of RONS, as well as for other cancer treatment methods that cause an increase in the concentration of extracellular RONS. Indeed, cancer cells contain less cholesterol than their healthy counterparts. Thus, they will be more vulnerable to the consequences of lipid peroxidation, eventually enabling the penetration of RONS into the interior of the cell, giving rise to oxidative stress, inducing pro-apoptotic factors. This provides, for the first time, molecular level insight why plasma can selectively treat cancer cells, while leaving their healthy counterparts undamaged, as is indeed experimentally demonstrated.

## Abstract 2

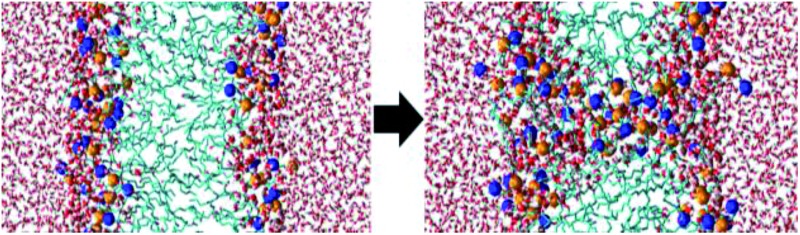
Molecular dynamics simulations suggest that the cholesterol-induced stability of lipid membranes during lipid peroxidation offers an explanation for the observed selectivity of plasma treatments towards cancer cells.

## 1. Introduction

The treatment of cancer has seen major progress over the last decades, but nevertheless, the current therapies are not always successful. In particular, the treatment selectivity towards cancer cells and the resistance of cancer cells against current therapies, are recurring issues.^1^ In recent years, a new therapy was developed for cancer treatment, based on cold atmospheric plasmas (CAP).^2–23^ A plasma is an ionized gas, which forms a highly reactive chemical cocktail, consisting of neutral species (molecules, radicals, excited species), ions, electrons and photons. Plenty of studies have already demonstrated that plasma is very promising for cancer treatment, for a large number of different cell lines, including breast cancer,^7^ lung cancer,^8^ gastric cancer,^9^ leukemia,^10^ pancreatic cancer,^11^ liver cancer,^12^ cervical cancer,^13^ melanoma,^13–15^ *etc.* Moreover, besides *in vitro* studies, several interesting *in vivo* studies have been presented.^16,17^ It is stated that plasma can selectively treat cancer cells, while leaving the healthy cells undamaged,^2,3,18,19,23–25^ and up to now, cancer cells have not yet developed resistance against plasma treatment.^2,3^ Furthermore, there is a synergistic effect of plasma treatment and traditional therapies. On the one hand, plasma treatment is able to make cancer cells that have developed resistance against traditional therapies, again susceptible for these traditional therapies.^20,21^ On the other hand, chemotherapy is able to make CAP-resistant cancer cells again sensitive towards this CAP-treatment.^26^ This is achieved through the regulation of the immune anti-oxidant system and the controlled intracellular production of anti-oxidant enzymes which scavenge ROS.^26^

However, in spite of these promising results, the underlying mechanisms of the plasma–cell interaction remain elusive. The most commonly accepted theory is based on oxidative stress, caused by the reactive oxygen species (ROS) formed in the plasma, when these species can enter the cancer cells. The same species are also thought to be responsible for apoptosis in cancer cells by the traditional cancer therapies.^27,28^ It is thus important to note that the observations presented in this paper are not merely limited to plasma cancer treatment, but can be expanded to other cancer therapies that deliver a sufficient amount of ROS to result in oxidative stress in the cell interior (*e.g.* radiation therapy or e-beam treatment). However, the emphasis is on plasma cancer treatment because, as mentioned above, this therapy has proven to be very selective towards cancer cells. One of the goals of this study is thus to find the underlying reason behind this observed selectivity. Moreover, besides ROS, reactive nitrogen species (RNS) produced by the plasma might also play an important role. The interplay between oxidative and nitrosative stress is thought to be responsible for the lack of resistance against plasma treatment, in contrast to traditional therapies.^29^

A few theories try to explain why cancer cells seem more vulnerable to plasma treatment than their healthy counterparts.^14,23^ One of them is based on the difference of the cell cycle of healthy and cancer cells. Because cancer cells divide much faster, there are more cancer cells in the S-phase (DNA-replication phase), which is thought to be the target phase of plasma treatment, explaining why especially cancer cells will be attacked by the plasma.^14^ Another theory for the selectivity of plasma treatment is related to the enhanced concentration of ROS and RNS found in cancer cells. The extra ROS and RNS produced by the plasma can increase the RONS concentration in the cells to such an extent that apoptosis occurs. Normal cells have much lower initial RONS concentrations, so they can handle the enhancement of the RONS concentration, by means of antioxidants.^30^ However, the way in which the ROS and RNS from the plasma are able to enter the cells cannot be explained by the above mentioned theories. Therefore, we hypothesize that the selectivity of plasma treatment towards cancer cells might arise from the alterations in the lipid composition of the plasma membrane of cancer cells. Indeed, it is known that the plasma membrane of cancer and healthy cells have a somewhat different composition. More specifically, cancer cells have a significantly lower concentration of cholesterol in their plasma membrane.^31,32^ In literature, for healthy cells, the molar ratio of cholesterol to phospholipids is estimated between 0.6 and 1.0,^31–33^ while for cancer cells, this ratio varies between 0.31 and 0.46.^31,32^ As cholesterol is mainly responsible for the ordering of the lipids in the plasma membrane,^34,35^ a lower cholesterol concentration increases the fluidity of the plasma membrane,^31,36^ making the membrane more vulnerable to the oxidative stress which arises from the impinging RONS. Although cholesterol metabolites (*e.g.* bile salts or vitamin D derivatives) also play an important role in membrane structures,^37,38^ these are not investigated in the present study.

Upon interaction with the plasma membrane, the RONS indeed cause peroxidation of the membrane lipids. This process has significant effects on the structure and dynamics of lipid membranes, such as an increase of the water permeability, a decrease of the bilayer thickness or alterations in the lipid membrane order and fluidity.^39–46^ Experimental studies focusing on the effect of lipid peroxidation on the membrane fluidity have, however, yielded contradictory results. While some researchers measure an increase of the membrane fluidity,^39–41^ others observe no alteration,^41^ or even a decrease of the fluidity.^43,44^ Several explanations are given to explain this contradiction, which include the sample preparation method or the depth to which the measuring probe enters the bilayer.^45^ Indeed, deep in the hydrocarbon core of the bilayer, the lipid order decreases after lipid peroxidation, while the region closer to the head groups is less affected. Thus, depending on the probe used, different results will be obtained.^47^

As mentioned above, in contrast to the destabilizing effect of lipid peroxidation products, cholesterol is known to exert a stiffening effect on a membrane. The question thus arises whether cholesterol can maintain its stabilizing effect in lipid membranes that contain lipid peroxidation products. In search of an answer, Megli *et al.* studied the alterations of the structure of oxidized bilayers upon addition of 40 mol% cholesterol.^48^ The results of these experiments show that the addition of cholesterol indeed ensures that the orientation of the bilayer lipids is maintained, even when the bilayers are fully oxidized. However, these experiments were only performed for 0 and 40 mol% of cholesterol, while cancer cells might contain a low, but non-negligible cholesterol fraction (see above).^31,32^ Moreover, it is difficult to obtain information on the molecular level from experimental studies. For this purpose, molecular dynamics (MD) simulations are extremely valuable, as they can study processes that occur on sub-nm length scales and on ps–ns time-scales. Therefore, in the present study, we perform MD simulations to gain atomistic insight in the effect of cholesterol, in different concentrations, on the membrane order and permeability upon lipid peroxidation, searching also for a threshold from which cholesterol is able to exert its protecting role. By linking our results to experimental observations of plasma cancer treatment, we provide a possible comprehensive theory to explain both the selectivity of cold atmospheric plasmas towards cancer cells, as well as their ability to effectively kill these cells.

There exist already some MD simulations in literature, studying the effect of lipid peroxidation on the properties of the lipid bilayer.^46,49–52^ It was found that a certain degree of oxidation yields water defects to occur in the bilayer. However, as mentioned above, no connection was made to the treatment of (cancer) cells by methods that cause this kind of oxidative stress, such as plasma cancer treatment. Moreover, none of these simulations studied the effect of cholesterol. Because the latter is essential to study the difference between cancer cells and healthy cells, and might be the origin of the selectivity of plasma treatment against cancer cells, this aspect is crucial. Therefore, for the first time, we study the effect of lipid peroxidation products, in the presence of cholesterol, on the lipid order and permeability of the plasma membrane on an atomic scale, by means of computational methods.

## 2. Description of the model systems

Although the plasma membrane consists of lipids and proteins, both contributing for about 50% to the mass of the plasma membrane, our model system only considers the lipids, as they determine the bilayer structure.

**Fig. 1:**
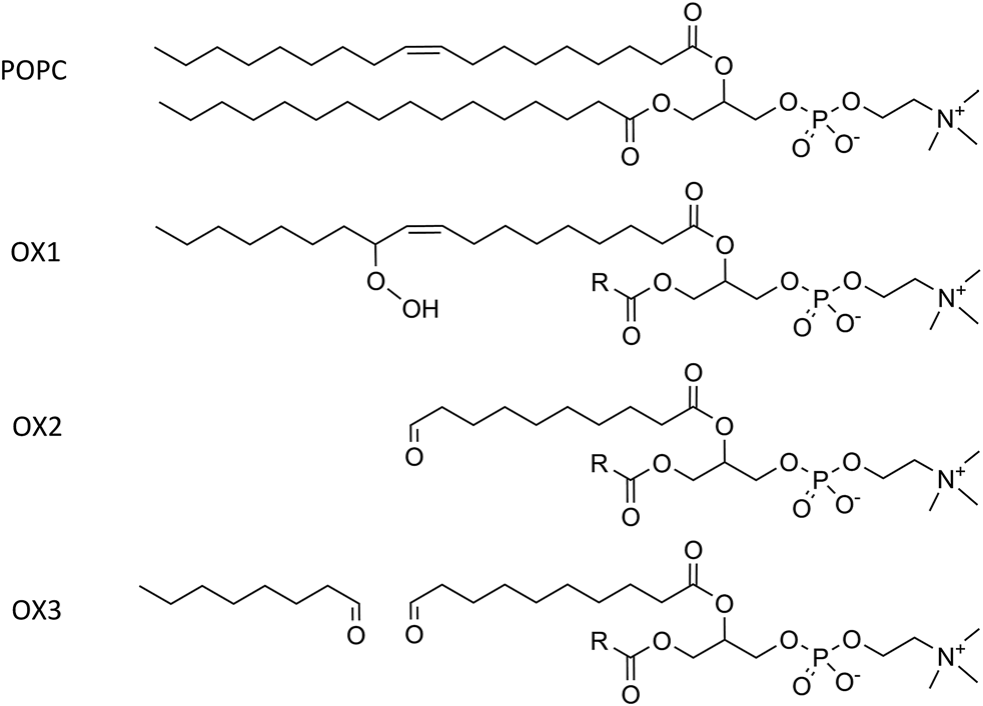
Illustration of a POPC molecule and the three oxidation products considered in this study. The R-group in the structures of OX1, OX2 and OX3 represents the native palmitoyl-chain.

The model systems constructed in this work each contain 72 lipids, more specifically 1-palmitoyl-2-oleoyl-*sn-glycero*-3-phosphatidylcholine (POPC) and cholesterol. POPC constitutes the largest fraction of the lipids in the plasma membrane. Besides non-oxidized plasma membranes, which contain only POPC and cholesterol, we focus especially on the presence of lipid peroxidation products in the membrane, as mentioned above. We therefore consider three representative oxidation products of POPC.^53^ These products are illustrated in Fig. 1, together with the normal POPC molecule. OX1 is formed by simple peroxidation of POPC, while OX2 and OX3 are formed by cyclisation of a peroxyl radical of POPC, followed by the breaking of this (unstable) ring structure, yielding 2 aldehydes. In OX2, only one aldehyde product is taken into account, while in OX3 both aldehyde products are considered, enabling to explicitly study the effect of the small aldehyde, which has never been done before.^49,54^

**Fig. 2:**
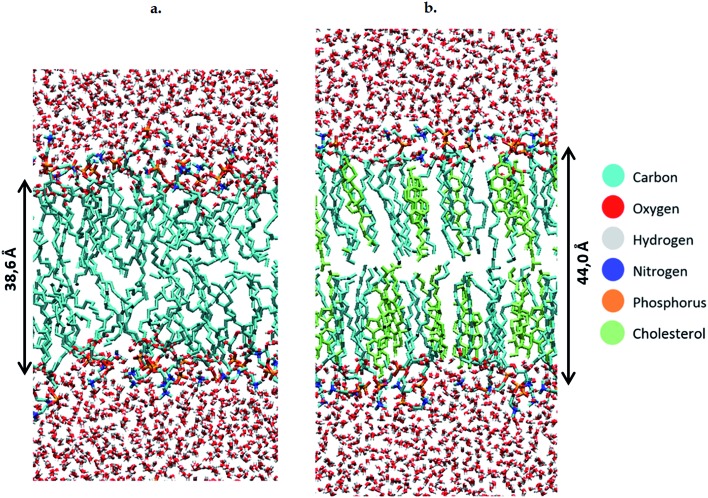
Structures of the POPC bilayer, without (a) and with (b) cholesterol.

To study the effect of these lipid peroxidation products, the concentration of either OX1, OX2 or OX3 is varied between 0 and 100%, by replacing a varying amount of POPC molecules by one of the oxidation products. Although a membrane will not become fully oxidized during cancer treatment, it is still useful to investigate high oxidation degrees in the model systems. This is firstly because the peroxidation products may accumulate locally and temporarily on a longer timescale, due to the polar nature of the aldehydes and peroxides in an apolar environment (hydrophobic core of the membrane). Secondly, it is useful because the peroxidation process is a chain reaction, which means that initially the peroxidation products will be created close to each other. It should also be noted that when replacing POPC by OX3, one phospholipid is being swapped for both oxidation products (both the phospholipidic as the short-chain aldehyde). However, further in this paper (*e.g.* in the calculation of the area per lipid, see below), these two oxidation products will be counted as one lipid, since they originate from one POPC molecule. We make these model systems for a plasma membrane containing 72 POPC molecules, as well as for bilayers containing a various number of cholesterol molecules (up to 50%). When creating the different model systems, POPC molecules are replaced, so that the total number of lipids (*i.e.*, the sum of either POPC, cholesterol, or one of the peroxidation products) still amounts to 72. In total, we constructed 56 model systems. Finally, besides the 72 lipids, each system also contains 4000 water molecules. Both lipids and water molecules are initially positioned in a box of about 5.5 × 5.5 × 11 nm^3^. An illustration of the model structure of the POPC bilayer, surrounded by water molecules at both sides of the lipid bilayer, is presented in Fig. 2(a). Fig. 2(b) illustrates the model system containing 36 POPC lipids and 36 cholesterol molecules.

## 3. Computational details

Non-reactive united-atom molecular dynamics simulations are applied to observe pore formation in the plasma membrane, occurring at a time-scale of several ns.^54^ In this approach, C-atoms and H-atoms bonded to it are treated as one group, *e.g.*, the methyl or methylene groups in the apolar tails of the phospholipids. In this way, one POPC molecule, containing 134 atoms, can be reduced to a system of 52 particles. Polar H-atoms, like in an alcohol-group or in the water molecules, however, remain as separate atoms, therefore allowing to study explicitly the effect of lipid peroxidation products.

We apply the GROMOS 43A1-S3 force field,^55^ since this force field contains parameters for cholesterol, in contrast to the force fields applied earlier to study the effect of lipid peroxidation. It also contains parameters for a wide variety of other lipids, but the peroxide and aldehyde groups, which occur in the oxidized lipids, were not yet included in this force field. Therefore, we had to implement them from literature.^49^ The parameters, together with the fitting procedure, can be found in Table S1.†

The 56 different model systems, outlined in Section 2 above, are constructed by randomly placing the various lipids in the simulation box, keeping the atoms of different lipids at a minimum distance of 2 Å from each other, to avoid unrealistically large forces at the start of the MD simulation. To generate this initial configuration, the Packmol package was used.^56^ After an energy minimization, using the steepest descent algorithm, MD simulations were run for 160 ns in the NPT ensemble (*i.e.*, constant number of particles, pressure and temperature), using a time-step of 2 fs. The first 80 ns of each simulation were considered as an equilibration period. During the simulation, the geometry of the system is recorded after each 100 ps, enabling us to average the output of the simulations for the further analysis (see further). We made use of the GROMACS 4.6 software for all simulations, as well as for analyzing the results.^57^ Furthermore, periodic boundary conditions were applied in all directions. A 1.0 nm cut-off was imposed for the electrostatic and van der Waals interactions. For the long-range electrostatics, we made use of the PME method,^58,59^ employing a 1.0 nm cut-off for real-space interactions and a 0.15 nm spaced grid for the reciprocal-space interactions, with sixth-order B-spline interpolation. To keep the temperature constant, the system is coupled to an external heat bath. We made use of the Nosé–Hoover thermostat, at a reference temperature of 303 K, and a coupling constant of 0.2 ps.^60^ The pressure is controlled by the semi-isotropic Parrinello–Rahman coupling scheme,^61^ with a reference pressure of 1 atmosphere, a compressibility of 4.5 × 10^–5^ bar^–1^, and a coupling constant of 1 ps.

## 4. Analysis of the properties of the bilayers

To investigate the effect of the lipid peroxidation products, we analyzed some typical properties of lipid bilayers, *i.e.*, the surface area per lipid, the thickness of the bilayer, the so-called deuterium order parameter and the water density at the center of the bilayer.

The surface area per lipid is calculated by dividing the surface area of the entire system (averaged over the final 80 ns simulation time, by sampling the data after every 100 ps) by the number of lipids present in one layer. To estimate the error bars, we applied the block method of Hess.^62^

The thickness of the lipid bilayer is determined from the distance between the phosphate groups of the two opposite layers of the bilayer system. To exactly localize the phosphate groups, the simulation box is divided into 100 intervals in the *z* direction, and the P atom density in each of these intervals is calculated, again by sampling after every 100 ps and averaging over the final 80 ns simulation time. The peak-to-peak distance between the two maxima in the P atom density profile is taken as the thickness of the lipid bilayer, with error bars determined from the width of the intervals.

The deuterium order parameter, *S*_CD_, is a measure for the order of the lipid tails in the bilayer. This parameter can also be determined by NMR, so that computational and experimental data can be compared. It is defined as follows:1
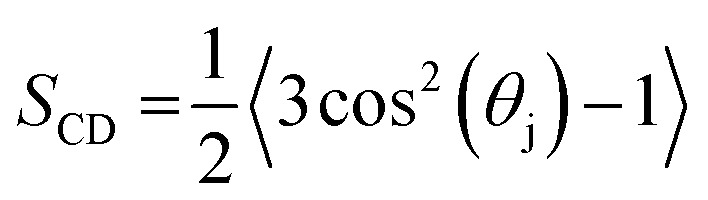
where *θ*_j_ is the angle between a C–D bond and the normal of the membrane (*z*-axis). If *S*_CD_ equals 1, the lipid tails are perfectly oriented along the *z*-axis, whereas lipid tails perpendicular to the *z*-axis would yield *S*_CD_ equal to –1/2. In the above formula, the average is taken over both C–D bonds of a CD_2_-group, for all lipids, as well as averaged over time. In the united-atom representation, no deuterium atoms are explicitly considered. Therefore, for the sake of this analysis, they were added to the C-atoms of the lipid tails, according to an ideal tetragonal symmetry. Although the deuterium order parameter is determined for each C-atom of the sn-1 chain, *i.e.*, the palmitoyl-chain that is not oxidized (see Fig. 1 above), the average is taken over all C-atoms, to be able to compare different systems.

The water density in the inner 1 nm of the bilayer, again averaged over the final 80 ns, is used as a measure for the polarity inside the membrane. The water density is calculated in the same way as explained above for the P-atom density.

## 5. Results and discussion

### 5.1. Validation of the force field

To validate the GROMOS 43A1-S3 force field for this application, we first calculated the surface area per lipid and the thickness of the bilayer, for a pure POPC bilayer, and we compared these calculated values with results from experimental or other computational studies from literature.^63–65^ From these data, which can be found in Table S2 of the ESI,† we can conclude that our computational results are within the range of data reported in literature. Our model can thus be used to investigate the effect of the addition of lipid peroxidation products and cholesterol, on the structural and dynamic properties of the phospholipid bilayer.

### 5.2. Influence of the concentration of lipid peroxidation products

First, we investigate the effect of the lipid peroxidation products in the model system without cholesterol, hence containing only POPC and one of the three peroxidation products mentioned in Section 2 above. The concentration of these peroxidation products is varied between 0 and 100%.

**Fig. 3:**
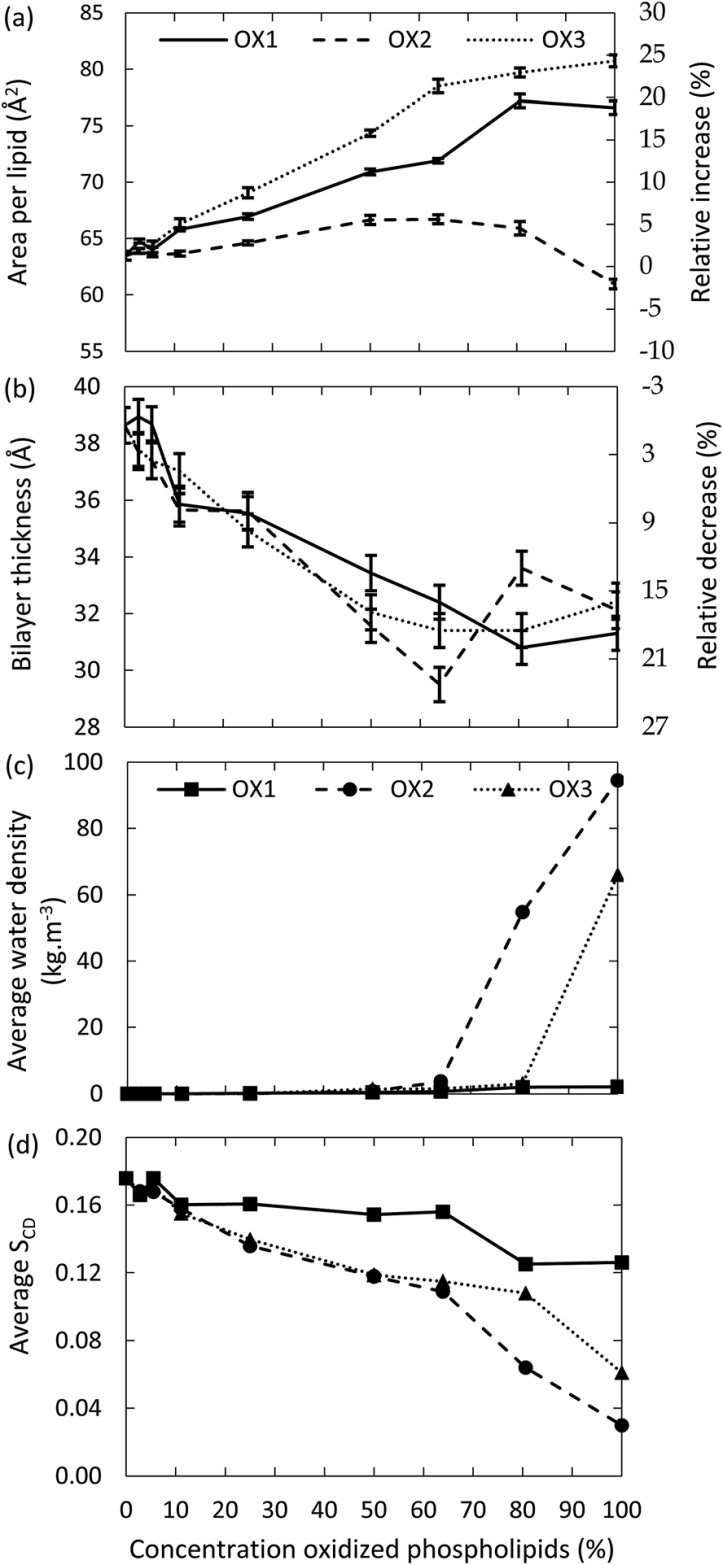
Surface area per lipid (a), thickness of the bilayer (b), average water density in the center of the bilayer (c) and average deuterium order parameter (d), as a function of the concentration of the oxidized phospholipids, for three types of oxidation products, for the model systems without cholesterol.

The surface area per lipid, thickness of the bilayer, water density inside the bilayer and deuterium order parameter for the model systems without cholesterol are plotted in Fig. 3, as a function of the concentration of the oxidized phospholipids, for the three types of oxidation products. It is clear that the surface area per lipid (Fig. 3(a)) generally increases upon oxidation. The right *y*-axis of Fig. 3(a) illustrates the relative increase, compared to the non-oxidized model system. Our results are in good agreement with experimental and computational observations from literature.^46,49,52^ For instance, at an oxidation degree of 50%, Wong-ekkabut also predicted a relative increase of the surface area of *ca.* 10%, for a similar oxidation product as OX1.^49^

**Fig. 4:**
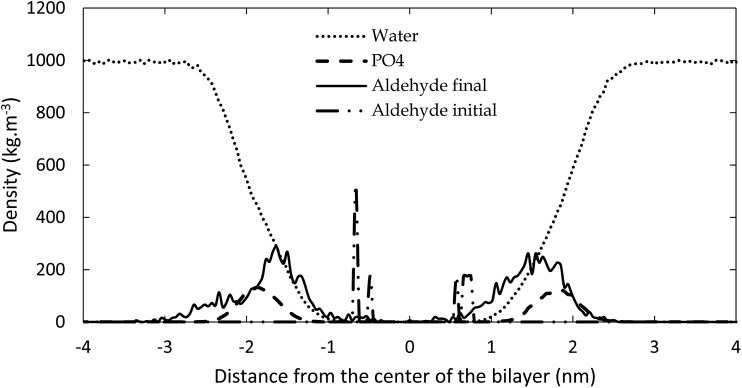
Densities of water, phosphate groups and aldehyde groups of OX3, both initially and during the final 80 ns of the simulation, as a function of distance from the center of the bilayer, for 11.1% oxidation.

The reason for the larger surface area upon oxidation is that the polar groups formed after oxidation prefer to move towards the water layer, to maximize their interactions with the water molecules. This bended conformation results in a larger surface area within the membrane, compared to a linear conformation of the non-oxidized lipid tails. This behavior is illustrated in Fig. 4, in which the density of the aldehyde groups of OX3 is plotted as a function of the distance to the center of the bilayer, for both the initial structure and the averaged structure over the last 80 ns of the simulation, in case of the 11.1% oxidized bilayer. The phosphate and water densities are also plotted, to indicate the position of the bilayer. Initially, the aldehyde groups are positioned halfway the bilayer, where the oxidation occurs, but in the course of the equilibration period, they bend towards the phosphate groups, where they can interact with the water molecules. As this is the most stable configuration, the aldehyde groups remain in this position during the entire simulation, as is clear from Fig. 4. This also means that the short-chain aldehyde products, present in OX3, remain stable in the bilayer. During the simulated timeframe, they were not seen to partition into the bulk of the surrounding water layers.

It appears from Fig. 3(a) that the increase in surface area per lipid is larger for OX3. This is most likely due to the presence of two shorter chains in this molecule, which can more easily change their conformation, due to a higher mobility. The peroxide (OX1), on the other hand, still contains two long apolar tails, which makes it more difficult for the polar group to alter the conformation of the lipid tails and thus to affect the structure of the bilayer. In the case of OX2, two competing effects occur. On the one hand, the structure will expand due to the movement of the polar groups towards the water layer, as is observed for the other oxidation products. On the other hand, OX2 does not contain the small aldehyde, as in OX3; hence, introducing OX2 in the bilayer yields extra free space, so the entire structure can shrink in the three dimensions. The behavior observed in Fig. 3(a) is the result of these two competing effects. By comparing the different curves of OX2 and OX3, it is clear that the small aldehyde largely affects the consequences of lipid peroxidation.

**Fig. 5:**
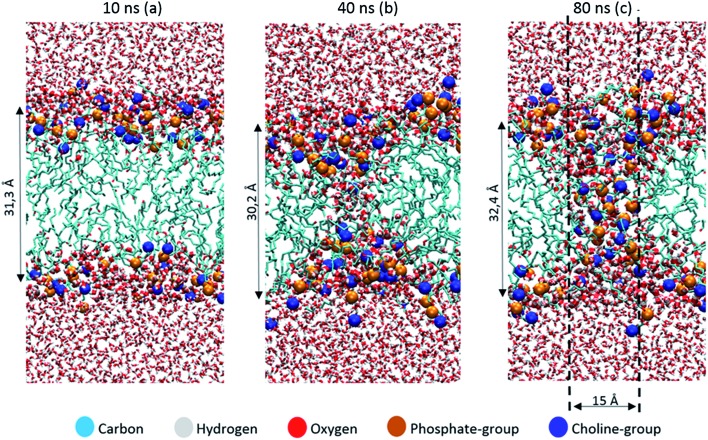
Snapshots of the MD simulation, after 10 ns (a), 40 ns (b) and 80 ns (c), illustrating the pore formation, in the model system without cholesterol and with 100% oxidation to OX3.

The thickness of the bilayer generally drops upon oxidation, as is clear from Fig. 3(b). This is again the result of the movement of the polar groups towards the water layer, which allows the two lipid layers to move towards each other. The relative decrease in thickness is indicated by the right *y*-axis. The decrease for OX3 is again slightly more pronounced than for OX1. For OX2, the two effects described above enhance each other in this case, explaining why the decrease is now even slightly more significant than for OX3. On the other hand, for oxidation approaching 100% in case of OX3, and 82% in case of OX2, the bilayer thickness rises again. This is attributed to pore formation, as is illustrated in Fig. 5 for OX3. Fig. 5(a) shows the initial conformation (after 10 ns), without water defects. After 40 ns (Fig. 5(b)), some water molecules have moved into the center of the bilayer, and after 80 ns (Fig. 5(c)), a pore is created. The thickness of the bilayer for the three time frames is also indicated in Fig. 5. Initially (*i.e.*, from (a) to (b)), the thickness still drops, due to the movement of the polar groups to the water layers, but when enough water molecules have moved to the center of the bilayer, the membrane swells, explaining the rise in thickness (c).

The pore diameter is at maximum about 15 Å. This is in agreement with,^51^ in which a pore with similar diameter was observed for the same oxidation product as OX3 (for DOPC instead of POPC) in case of 100% oxidation. Moreover, Leduc *et al.* observed this pore formation process experimentally during plasma treatment of HeLa cells, which they assigned to either the lipid peroxidation process or cell charging.^66^ Since we expect the pore size observed in our simulations to be too small for entire organelles or macromolecules to travel through the membrane, this observation offers a possible explanation why typically apoptosis instead of necrosis is observed during plasma treatment of cancer cells.^19^ Plasma species, like RONS, on the other hand, are small enough to travel through these pores, so this means they can reach the interior of the cell, where they can react with biomolecules such as DNA or proteins.

The fact that pore formation already occurs for a lower oxidation degree in case of OX2 can be explained by the lower thickness of the bilayer at 63% oxidation, *i.e.*, just before pore formation occurs (see Fig. 3(a)). Due to the thinner bilayer, water molecules can more easily reach the center of the bilayer. In systems containing OX1, the thickness of the bilayer also drops upon oxidation, but within the simulated time scale, no pore formation was observed.

The latter can also be deduced from Fig. 3(c), illustrating the water density in the center of the bilayer upon oxidation, for the three oxidation products. It is clear that the water density is negligible in case of oxidation degrees up to 60% (in case of OX2) and 80% (in case of OX3), but a higher oxidation degree leads to a significant rise, corresponding to the pore formation. In case of OX1, the water density remains very low, even up to 100% oxidation, indicating again that no pore formation occurs.

Finally, the deuterium order parameter is plotted against the oxidation degree in Fig. 3(d). A decrease in the order of the lipid tails is observed, due to the distortion caused by the polar groups in the apolar environment. The effect is again more pronounced for the aldehydes (OX2 and OX3), because of the shorter chain lengths. In general, we can conclude that upon oxidation, the membrane order decreases, which can eventually lead to pore formation.

### 5.3. Influence of cholesterol on the effect of lipid peroxidation

The results shown above were obtained for model systems that do not contain cholesterol. Besides these, we also studied model systems containing 36 cholesterol molecules and a varying concentration of POPC and peroxidation products. Again, the concentration of these peroxidation products is varied between 0 and 100%. Note that we make here the approximation that cholesterol is not oxidized during the plasma treatment. We can justify this approximation, since peroxidation of cholesterol is much slower than peroxidation of unsaturated phospholipids.^67^ This enables us to safely assume that a large fraction of the phospholipids is oxidized during plasma treatment, without significantly affecting the structure of cholesterol. Moreover, the oxidation products of cholesterol still contain rigid ring structures, so we can expect that even upon oxidation, the effect on the structure of the bilayer would be limited.

Before studying the effect of the oxidation products, we have compared the calculated surface area per lipid, thickness of the bilayer and average deuterium order parameter for the non-oxidized POPC bilayer, with and without cholesterol (see Table S3 in the ESI†). Upon addition of cholesterol, the surface area per lipid significantly drops. The reason for this is twofold. First, the calculations for the surface area per lipid also account for cholesterol as a lipid, and as the latter is much smaller than a phospholipid (*i.e.*, surface area per cholesterol molecule is estimated at 22 Å^2^ (ref. 68)), the average value drops. Furthermore, as mentioned before, due to the rigid rings, cholesterol is known to exert a stiffening effect on the lipids of the membrane. This stiffening effect is characterized by a higher ordering of the bilayer, which can also be deduced from the higher average deuterium order parameter. Finally, the addition of cholesterol yields a thicker bilayer, due to the more stretched conformation of the lipid tails, as could also be observed from Fig. 2 above.

The surface area per lipid, thickness of the bilayer and deuterium order parameter, for the model systems with cholesterol, as a function of the concentration of the oxidized phospholipids, for the three types of oxidation products, are illustrated in Fig. S1 of the ESI.† In general, a similar behavior is observed as for the systems without cholesterol. Fig. 1(a) indicates a rise in surface area for OX1 and OX3, and a small decrease for OX2. The latter can again be explained as the result of the two competing effects (see Section 5.2 above). The effect of the extra free space is more pronounced in this case, as cholesterol ensures that the ordered structure makes optimal use of this free space, leading to a drop in the surface area.

The thickness of the bilayer again generally drops upon oxidation, but in contrast to the systems without cholesterol, no increase is observed upon 100% oxidation. This indicates the absence of pore formation when cholesterol is present. At most, only a few water molecules are able to penetrate in the bilayer (see Fig. S2 of the ESI†). Therefore, the average water density in the center of the bilayer remains close to zero (*i.e.*, at maximum 1.3 kg m^–3^).

Finally, the trend of the average deuterium order parameter is again identical to the observed trend for model systems that do not contain cholesterol, as appears from Fig. S1(c).† Although a decrease in order is observed, the final values (for 100% oxidation of the phospholipids) are still larger than the value obtained for the system with only POPC (*i.e.*, 0.176; see Table S3 in the ESI†). This indicates that, in spite of the disorder created upon oxidation, the cholesterol molecules still ensure that the bilayers are more strictly ordered than in the case of the pure POPC bilayer. This stronger ordering is responsible for the absence of pore formation in the systems containing cholesterol.

Our results thus confirm the experimental results obtained by Megli *et al*.^46^ However, we want to couple these results to the observed selectivity of plasma treatment towards cancer cells. Since the cholesterol to phospholipid ratio of the plasma membrane of cancer cells is estimated to be around 0.3–0.46,^31,32^ lipid bilayers with no cholesterol at all are only a rough approximation. Therefore, we also varied the cholesterol concentrations between 0 and 50%. We focus here on the case of 100% oxidation to OX3. OX3 was chosen because no pore formation was observed in the case of OX1, and the behavior of OX2 was the result of two competing effects, making the observed trends more difficult to interpret.

### 5.4. Influence of the cholesterol concentration

**Fig. 6:**
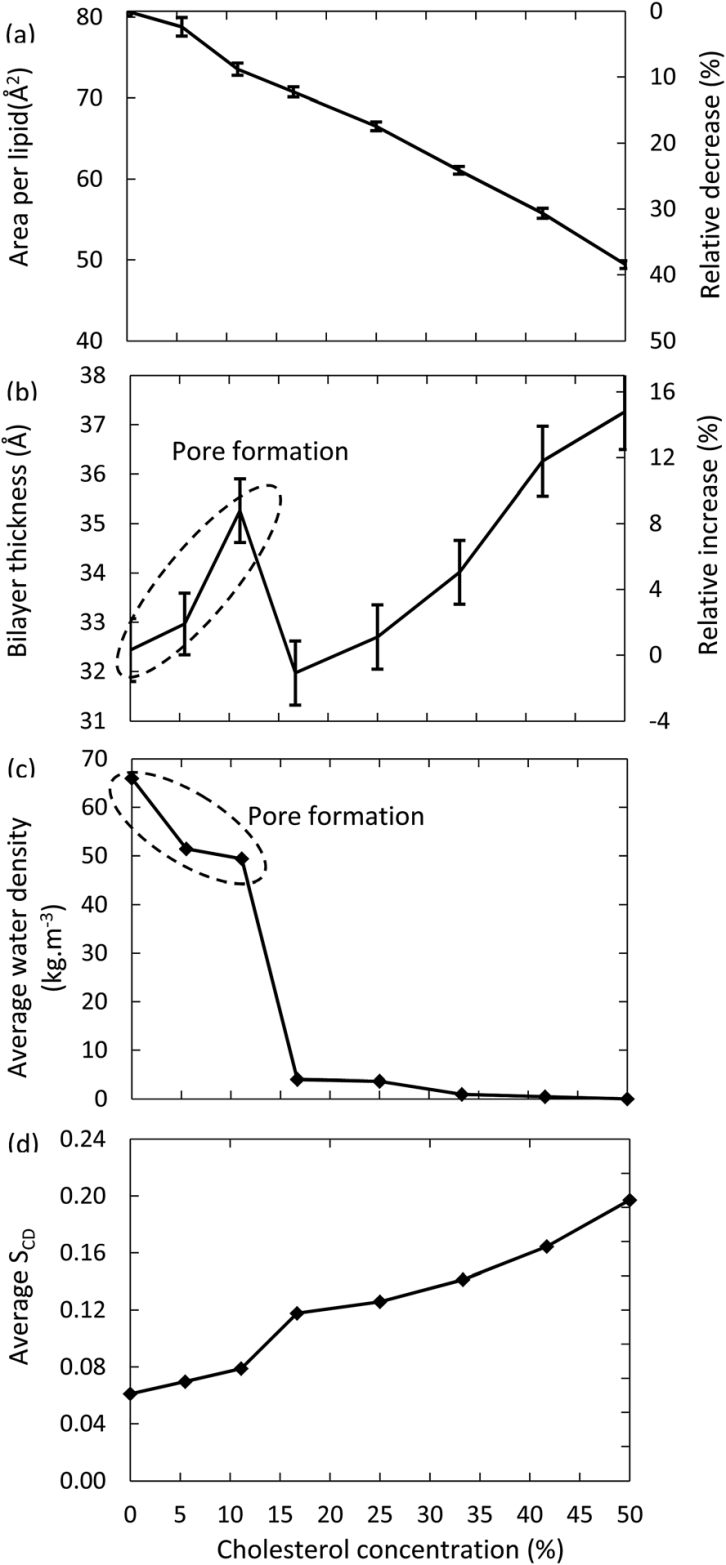
Surface area per lipid (a), thickness of the bilayer (b), average water density in the center of the bilayer (c) and average deuterium order parameter (d), for the model systems with 100% oxidation to OX3, as a function of cholesterol concentration in the bilayer.

Fig. 6 illustrates the surface area per lipid, thickness of the bilayer, average water density and deuterium order parameter, for the models system with 100% oxidation to OX3, as a function of the concentration of cholesterol.

The surface area per lipid (Fig. 6(a)) significantly decreases upon increasing cholesterol concentration, due to (i) the higher order, and (ii) the lower surface area taken by the cholesterol molecules. The thickness of the bilayer (Fig. 6(b)) first increases up to a cholesterol concentration of 11%, again due to the higher ordering, leading to a more stretched conformation of the apolar tails. However, a further increase of the cholesterol concentration yields a dramatic drop in thickness of the bilayer. The reason is that bilayers up to 11% of cholesterol still give rise to pore formation, while this appears impossible for lipid bilayers that contain higher cholesterol concentrations. As a result, the number of water molecules in the center of the bilayer drops drastically, as can also be deduced from Fig. 6(c). Hence, this explains the drop in bilayer thickness. When the cholesterol concentration rises further, the bilayer thickness increases again, due to the higher ordering, as explained above. Finally, Fig. 6(d) confirms the higher ordering upon increasing cholesterol fraction. The small jump observed between 11% and 16% cholesterol is again correlated to the pore formation behavior, as the absence of pores leads to a higher ordered bilayer structure.

The differences observed in the model systems without and with cholesterol, and the behavior as a function of increasing cholesterol concentration, are crucial in the framework of plasma for cancer treatment. Indeed, these results strongly suggest that the plasma membrane of cancer cells, which are supposed to contain very little cholesterol,^30^ is more prone to the consequences of lipid peroxidation, compared to the plasma membrane of healthy cells. Indeed, the lipid order decreases while the membrane permeability increases, eventually even leading to pore formation, allowing RONS from the plasma to reach the interior of the cell, where they can damage among others the DNA and proteins of the cancer cells. Healthy cells, however, are expected to be less sensitive to the consequences of lipid peroxidation, due to the presence of a higher cholesterol concentration in the plasma membrane. This indicates that they would not be affected by the plasma treatment. While other factors, such as the higher steady-state RONS concentration of cancer cells, and a higher fraction of cancer cells in the S-phase, are likely to be of importance as well, our modelling efforts for the first time offer a molecular level explanation for the high selectivity of plasma treatment towards cancer cells, while leaving their healthy counterparts unharmed, which has indeed been clearly demonstrated experimentally.^2,3,18,19,23–25^

## 6. Conclusion

We applied united-atom MD simulations to study the effect of lipid peroxidation on the properties of a POPC bilayer. Three different types of oxidized phospholipids were implemented, containing either a peroxide or aldehyde group as a result of the peroxidation reaction. We studied phospholipid bilayers without cholesterol and with cholesterol, as model systems for the plasma membrane of cancer cells and healthy cells, respectively, and we also varied the cholesterol concentration to elucidate its effect on the membrane order.

Our results clearly indicate that the phospholipid bilayer becomes more disordered as a result of the oxidation products. The effect is however more pronounced in the model systems without cholesterol, where pore formation was observed upon 100% oxidation of the phospholipids. These pores allow reactive species, such as RONS, to penetrate the plasma membrane, giving rise to oxidative stress inside the cell, inducing pro-apoptotic factors. Due to the small scale, the observed modifications (*e.g.* porosity) will, however, not affect normal receptor functioning or cause a disruption of intracellular signaling. When adding cholesterol in concentrations above 11% to the bilayer, pore formation does not occur. Indeed, as the plasma membrane of cancer cells contains significantly lower amounts of cholesterol,^31,32^ our simulations predict that cancer cells are more vulnerable to cancer treatments that deliver ROS externally, such as plasma cancer treatment. Indeed, these results are applicable to any cancer treatment therapy that delivers intra- or extracellular RONS. However, they are specifically extremely important for plasma treatment of cancer cells, since during this therapy, initially only extracellular RONS are generated. They should thus pass the plasma membrane to cause oxidative stress in the interior of a cell.

Although this study describes only a small fraction of the processes that occur during cancer treatment, we believe that our results contribute to a better understanding of the reasons that underlie the experimental observations. Indeed, as we demonstrate how extracellular RONS are able to penetrate model systems of the plasma membrane of cancerous cells, but not the plasma membrane of normal cells, cancerous cells thus can be selectively damaged while the healthy cells are left far less affected. This is indeed clearly demonstrated by various experimental studies,^2,3,18,19,23–25^ and thus, our simulations provide for the first time molecular level insight in this important aspect of plasma selectivity for cancer treatment.
