# Supplementary material for: Effect of lipid peroxidation on membrane permeability of cancer and normal cells subjected to oxidative stress
Source: Chem Sci. 2015 Oct 16;7(1):489–98. doi: 10.1039/c5sc02311d (PMC5518669; doi:10.1039/c5sc02311d)
Supplement: Supplementary file 1 [file SC-007-C5SC02311D-s001.pdf]

Electronic Supplementary Material (ESI) for Chemical Science.
This journal is © The Royal Society of Chemistry 2015

Supporting information

Table S1: Parameters used in the force field for describing the aldehyde and peroxide groups in the lipid bilayer, taken from [1]. The functional form for the dihedral angles is: E = a(1+cos(x-b)) + c(1+cos(2x-d)) + e(1-cos(3x-f)). Columns indicated by * are in kJ.mol^{-1}, columns indicated by † are in degrees.

Bonds		
Bond	r_{ij} (nm)	K_{ij}^{b} (kJ.mol^{-1}.nm^{-2})
C-O (peroxide)	0,14180	225670
O-O (peroxide)	0,14430	269580
O-H (peroxide)	0,09810	44130

Angles		
Angle	α_0 (graden)	K_{ijk}^{θ} (kJ.mol^{-1}.rad^{-2})
=C-C-O (peroxide)	104,00	418,40
C-C-O (peroxide)	109,50	418,40
C-O-O (peroxide)	105,90	598,37
O-O-H (peroxide)	100,00	506,92

Dihedral angles						
Dihedral angle	a*	b†	c*	d†	e*	f†
C=C-C-O (peroxide)	2,12	223,90	0	0	3,62	180,50
C-C-O-O (peroxide)	2,13	334,25	0	0	7,04	8,10
C-O-O-H (peroxide)	8,46	23,30	6,51	18,40	0	0
C-C-C-=O (aldehyde)	0,47	180,00	1,58	180,00	2,67	180,00

Partial charges				
Functional group	CH	O	O	H
Hydroperoxide	0,30	-0,30	-0,45	0,45
Aldehyde	0,53	-0,53	-	-

The parameters were derived using quantum chemical calculations, with the B3LYP method of density functional theory [2,3] and the LACV3P**++ basis set [4]. Partial atomic charges were estimated using natural population analysis [5] and the electrostatic surface potential fitting method with Merz-Kollman atomic radii [6] after the geometry optimization. For the calculation of bond and angle force constants, we restrained the bond lengths and angles at seven different values, then fitted a harmonic potential function to the energy profile. For dihedral parameters, dihedral angles were restrained at

36 different values from 0 to 360°, and the standard proper dihedral function was fitted to the potential energy. For all bonded parameters, the Lennard-Jones and electrostatic energy were calculated for different geometries and subtracted from the total energy before fitting.

Table S2: Comparison between our calculated data and experimental data from literature, for a pure POPC bilayer.

	Calculated value	Experimental data from literature [7-9]
Surface area per lipid (Å^2)	63.5 ± 0.4	63.0 – 64.3
Thickness of the bilayer (Å)	38.6 ± 0.6	37.5 – 39.1

Table S3: Calculated values for the POPC bilayer, without and with cholesterol (concentration of 50%).

	POPC	POPC/Cholesterol
Surface area per lipid (Å^2)	63,5 ± 0,4	42,1 ± 0,1
Bilayer thickness (Å)	38,6 ± 0,6	44,0 ± 0,6
Average S_{CD}	0,176	0,347

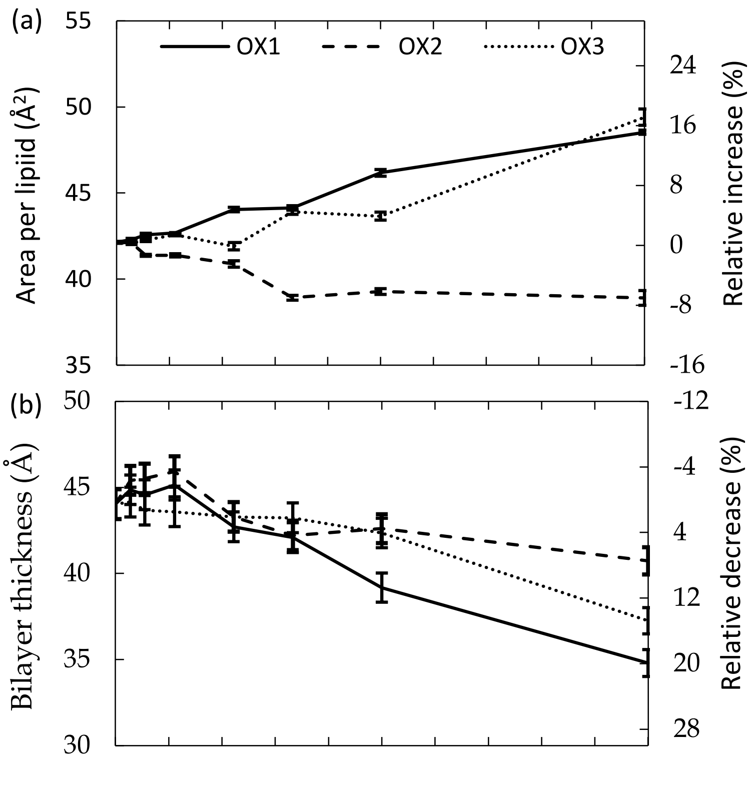

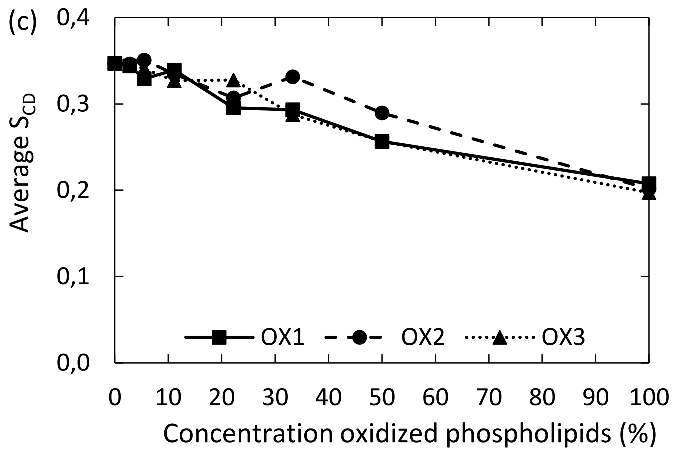

Figure S1: *Surface area per lipid (a), thickness of the bilayer (b) and average deuterium order parameter (c), as a function of the concentration of the oxidized phospholipids, for three types of oxidation products, for the model systems with 50% cholesterol.*

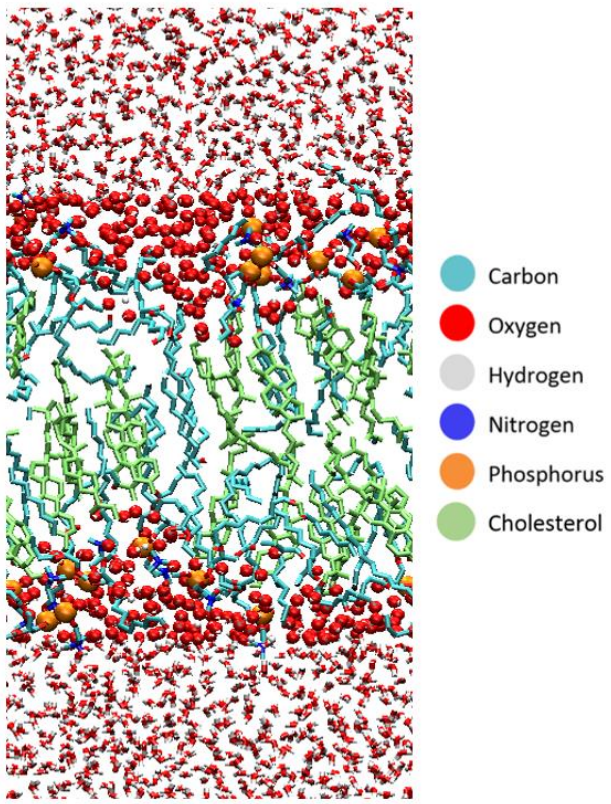

Figure S2: *Structure of the bilayer with 50% POPC and 50% cholesterol, and 100% oxidation of the phospholipids, showing that no pore formation occurs.*

References

1. J. Wong-Ekkabut, Z. Xu, W. Triampo, I-M Tang, D.P. Tieleman and L. Monticelli, Biophys. J., 2007, 93, 4225
2. I. Schrodinger, JAGUAR 4.1, 2000, Schrodinger Inc., Portland OR
3. A.D. Becke, *J. Chem. Chem. Phys.*, 1993, **98**, 5648
4. C.T. Lee, W.T. Yang and R.G. Parr, *Phys. Rev. B.*, 1988, **37**, 785

5. A.E. Reed, R.B. Weinstock and F. Weinhold, *J. Chem. Phys.*, 1985, **83**, 735
6. U.C. Singh and P.A. Kollman, *J. Comput. Chem.*, 1984, **5**, 129
7. N. Kučerka, M.P. Nieh and J. Katsaras, *Biochim. Biophys. Acta - Biomembr.*, 2011, **1808**, 2761
8. B.A. Lewis and D.M. Engelman, *J. Mol. Biol.*, 1983, **166**, 211
9. S. Leekumjorn and A.K. Sum, *J. Phys. Chem. B*, 2007, **111**, 6026
